# Exploring the Relationship Between Mental Fatigue and Injury Occurrence in Sport: Preliminary Evidence from a Male Semi-Professional Basketball Team

**DOI:** 10.3390/sports14040148

**Published:** 2026-04-10

**Authors:** Pierpaolo Sansone, Suzanna Russell, Carlotta Longo, Damiano Polverari, Bart Roelands

**Affiliations:** 1 Department of Education and Sport Sciences, Pegaso Telematic University, 80132 Naples, Italy; 2 Human Physiology and Sports Physiotherapy Research Group, Faculty of Physical Education and Physiotherapy, Vrije Universiteit Brussel, 1050 Brussels, Belgium; suzanna.russell@vub.be (S.R.); bart.roelands@vub.be (B.R.); 3 Department of Surgical Sciences, Università degli Studi di Torino, 10124 Turin, Italy; carlotta.a.longo@gmail.com; 4 PhD Program in Sport Sciences, Universidad Catolica San Antonio, 30107 Murcia, Spain; dpolverari@alu.ucam.edu; 5 Laboratory of Sports and Nutrition Research, Riga Stradiņš University, LV-1007 Riga, Latvia

**Keywords:** fatigue, injury risk, team sports, athlete monitoring

## Abstract

Mental fatigue (MF) has been hypothesized to contribute to injury risk in athletes, but observational studies have not directly investigated this relationship. Therefore, the current study evaluates potential relationships between mental fatigue and subsequent injury occurrence in basketball. Using an observational design, we monitored fourteen male semi-professional basketball players (age: 22 ± 4 years; stature: 192.6 ± 8.8 cm; body mass: 85.5 ± 9.1 kg; Tier 3) from a single team for 21 weeks throughout the competitive season. Each week, the players participated in 5 team-based training sessions, 2–4 individual training sessions, and 1–2 official games. Subjective MF ratings were collected using 100 mm visual analogue scales twice a week (the day before and after the official game) and then averaged. Time-loss injuries were registered, noting the body location, mechanism, and context (training and games). Generalized logistic mixed models were employed to evaluate whether MF levels were associated with injury occurrence in the subsequent 1, 3, and 5 days and 1, 2, 3, and 4 weeks of basketball activity. A total of 11 injuries were registered during the study (7.40 per 1000 h of basketball activity), with an average time loss of 12 ± 19 days. There were no associations between MF and injury occurrence in the following 1, 3, 5 days nor 1, 2, 3, 4 weeks (all *p* > 0.05, odds ratios: 1.00–1.28). In male semi-professional basketball settings, preliminary evidence indicates that MF might not be associated with injury occurrence. However, due to the dearth of injury events, the statistical power of this study is insufficient to detect potential small–medium effects. Therefore, the current results should be considered exploratory as opposed to a definitive rejection of the hypothesis. Future studies should evaluate the relationship between MF and injury risk in larger samples and among professional athletes.

## 1. Introduction

Injuries are a negative outcome of practicing sports that can have serious consequences, such as emotional and mental distress [1], performance consequences, and high financial costs [2,3]. Due to its high intensity [4], the physical proximity between players, and frequent contact [5], basketball, an invasion-based team sport, poses a high risk of injury. Professional players incur 12.6–21.7 injuries per 1000 h of basketball exposure [2,3,6], with increased rates during competitive games [2,3]. This incidence may be subject to further increases given the accelerating pace of the game [7] and schedule congestion [8] observed in contemporary competitive basketball [2,6], implying greater external loads and intensities imposed on players, further potential risk factors for injuries [9]. It is therefore of interest to the basketball population to identify factors associated with injuries and strategies to reduce their likelihood of occurrence and severity.

Mental fatigue (MF), a psychobiological state induced by demanding cognitive activity characterized by tiredness, lethargy, and a lack of motivation [10], has been receiving an increasing amount of attention from sport researchers in recent years. In fact, MF has been shown to negatively affect several domains of sport performance, including endurance [10], accuracy, and decision-making [11]. While this evidence comes from laboratory-based experimental designs, MF is a naturally occurring phenomenon [12] in team sports. In fact, during training and games, players must perform at their best for extended periods, scanning their dynamic environments, exerting near-maximal efforts, and undergoing frequent decision-making [13,14]. Repeated exposure to these mentally demanding scenarios during training and [15] competition could induce MF and in turn affect basketball players not only acutely but also cumulatively. As suggested in recent studies [16,17], elite sport practitioners report that cumulative exposure to training and competition without adequate mental recovery is a common perceived cause of MF, evincing the cumulative feature of MF manifestation. Athletes and elite practitioners describe the subjective experience of MF as a complex state characterized by cognitive, emotional and motivational symptoms [15,16]. Furthermore, basketball players perceive that several sport-specific factors can induce MF, such as individual perceptions of performance and training and recovery scheduling [15]. Thus, MF is likely to manifest throughout the competitive sport season, as found in previous studies [18,19,20].

It has been postulated that MF may also negatively impact injury occurrence. Schampheleer and Roelands [21] hypothesized that a mentally fatigued athlete might be at greater risk of injury due to delayed response times, suboptimal decision-making, and changes in motor control and biomechanical patterns, potentially leading to risky scenarios during competition, especially during physical contact. These hypotheses evolved from previous studies showing how MF worsens balance, postural control, and neuromuscular activation in healthy adults [22,23] and response times, accuracy, and decision-making in athletes [11]. Furthermore, professional sport practitioners (including managers, coaches, and medical staff members) perceive MF as a factor that may increase the risk of injuries in invasion sports [14]. For example, the study by Fazackerley et al. demonstrated that practitioners feel that mentally fatigued athletes might be more susceptible to injuries, especially non-contact ones, and that MF is linked to injury risk due to its negative effects on movement control, decision-making, focus, and task (dis)engagement [14]. However, to the best of our knowledge, no studies have quantitatively explored the potential associations between MF and injury occurrence in sports. Accordingly, the aim of this study was to explore the potential relationship between MF and injury occurrence through the observation of male basketball players during the competitive season. The hypothesis of this work was that there is a small association between MF and injury occurrence.

## 2. Materials and Methods

### 2.1. Experimental Approach to the Problem

This study aimed at directly addressing whether there is an association between MF and injury occurrence in team sport athletes. While this relationship was hypothesized in earlier work [21] and is perceived to exist among elite sport practitioners [14], no previous studies systematically investigated this relationship. Therefore, in this work, we implemented a prospective longitudinal observational cohort study with repeated weekly measures, together with injury surveillance, to capture the players’ perceptions of MF during an official competitive season of basketball.

### 2.2. Participants

Fourteen adult male basketball players (age: 22 ± 4 years; stature: 192.6 ± 8.8 cm; body mass: 85.5 ± 9.1 kg) volunteered to participate in this study. All players belonged to the same team, which competed in an interregional basketball league (Tier 3) [24]. Players typically participated in 4–5 team-based basketball training sessions, 2–4 individual physical training sessions, and 1–2 official matches each week. This sample was purposedly selected as being representative of the population of interest (i.e., adult male competitive basketball players). The monitoring window covered 21 consecutive weeks during the competitive basketball season. All players continuously participated in the team and research activities for the entire duration of the study. The study was approved by the Institutional Review Board of the Pegaso Telematic University (#001218). Players provided informed written consent prior to participation.

### 2.3. Mental Fatigue Monitoring

**Figure 1:**
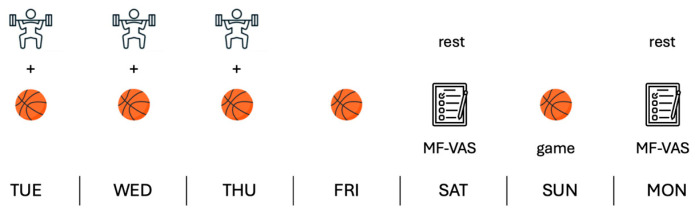
Typical microcycle organization of the team monitored.

Prior to commencement of data collection, an experienced sports scientist educated the participants about MF. In this explanation, MF was defined as the feelings of tiredness, reduced energy levels, and lack of motivation induced by prolonged cognitive activity [10], differentiating it from physical fatigue. Subjective MF ratings were collected using electronic visual analogue scales (0 = no mental fatigue; 100 = extreme mental fatigue) twice weekly by means of a smartphone, following previous standards used in elite-athlete MF monitoring [18,25]. The questionnaire specifically gave the athletes the following instruction: “please report your level of MF at this moment.” Questionnaires were administered in the morning (at 09.30 AM) on the rest day before an official game (match day minus one, MD-1) and on the rest day after a game (match day plus one, MD+1) (Figure 1). If players did not complete the questionnaire within 30 minutes, a reminder was sent to encourage compliance. MD-1 was selected to reflect the MF induced by the weekly training load, while MD+1 is more reflective of competition-induced fatigue [19,20]. At the same time, we tried to limit the burden imposed on the athletes by excessive monitoring procedures [26]. The two scores were averaged to obtain a unique value per week, which was informative with respect to the MF experienced by players alongside both training and competition demands. In case of missing responses (between MD-1 and MD+1), only one value was considered for analysis. The compliance rate over the two time-points was very good (82%).

### 2.4. Injury Surveillance

A record of the injuries sustained by athletes was completed daily by the medical staff of the team, who were qualified and experienced with injury surveillance procedures. Following previous research standards [27,28], we defined injuries as instances where a player was unable to fully participate in basketball training or games due to any physical complaint [27,28]. For each injury, the date, context (training or game), location (head/face; neck/cervical spine; shoulder/clavicula; upper arm; elbow; forearm; wrist; hand/finger/thumb; sternum/ribs/upper back; abdomen; lower back/pelvis/sacrum; hip/groin; thigh; knee; lower leg/Achilles tendon; ankle; and foot/toe), typology (bone; ligament/joint; muscle/tendon; and contusion), mechanism (traumatic or atraumatic), and date of return to team activities were registered to calculate the time loss in days [3,27]. Each injury was considered a new event for players who suffered from different injuries throughout the 21-week period. Illnesses were not considered sport-related injuries. Athlete exposure was calculated as the duration (in hours) corresponding to both training activities and games [3,28]. Injury incidence was calculated as the number of total injuries divided by the total exposure (hours of basketball activity) and reported as the rate of injury per 1000 h of basketball activity [3,28].

### 2.5. Statistical Analyses

Statistical analyses were performed with Jamovi (version 2.3, Jamovi Project, Sydney, Australia). Generalized logistic mixed models were computed, with MF inserted as a covariate (continuous variable), players’ ids inserted as a random effect (to account for repeated measures), and injury occurrence selected as a categorical (yes or no) dependent variable. Multiple separate models were run to evaluate whether MF levels were associated with injury occurrence in the subsequent 1, 3, and 5 days as well as in the subsequent 1, 2, 3, and 4 weeks from MF data collection. These time frames were selected in consideration of the fact that MF can impact athletes both acutely [10] and cumulatively [16,17] and in view of previous findings showing that injuries can manifest up to four weeks after a change in an athlete’s status [29,30]. For each model, Akaike Information Criterion, R^2^ marginal, estimate, odds ratios, z and *p*-values, and confidence intervals were reported. As multiple models were tested, Holm–Bonferroni correction was applied by dividing the α level of 0.05 by the total number of tests minus the rank of each of the *p*-values.

## 3. Results

Over the 21 weeks of this study, six players suffered 11 injuries (5 of which were recurrent), with an occurrence rate of 7.40 injuries per 1000 h of athlete exposure. Ten injuries (91%) occurred during training, and the remaining injury (9%) occurred during a game. The locations of the injuries were as follows: five (45%) corresponded to the lower back/pelvis/sacrum, three (27%) affected the hip/groin, two (18%) affected the foot/toe, and one (9%) affected the ankle. Eight (73%) injuries were of an atraumatic nature, while three (27%) were traumatic. The average time loss for the registered injuries was 12 ± 19 days.

The average MF across the study was 44.1 ± 18.9 (VAS score), collected across a total of 482 out of the 588 questionnaires sent (compliance rate: 82%). Table 1 presents the outputs from the logistic mixed model analyses. While MF was neither associated with injury occurrence within 1, 3, or 5 days nor in the subsequent 1, 2, 3, or 4 weeks of basketball activity (all *p* > 0.05), the small sample size might have limited the statistical power for this study.

## 4. Discussion

This study examined the relationship between MF and injury occurrence in male semiprofessional basketball. The main finding of this study is that the subjective MF reported by athletes was not significantly associated with injury occurrence in the subsequent days or in the following 4 weeks of basketball practice. However, confidence intervals were wide, indicating substantial uncertainty.

To our knowledge, this is the first study investigating, through observational means, whether there is a relationship between MF and injury occurrence in sport. Previous authors [21] have hypothesized that there is a link between MF and injury risk based on laboratory findings showing reductions in postural control, neuromuscular activation, and decision-making in mentally fatigued subjects [11,22,23]; furthermore, sport practitioners (coaches, physiotherapists, and managers) see MF as an injury risk factor [14]. However, the results from our preliminary project do not support this hypothesis. It is important to note that injury risk among athletes is multifactorial and complex [31]. Studies have determined that several factors interact, in a non-linear fashion, ultimately influencing injury occurrence for an athlete. Characteristics such as sex [32], competitive level [2,6], previous injuries [32,33], physical qualities [31,33] (e.g., anthropometry, neuromuscular qualities, biomechanics, and muscle imbalances), psychology (e.g., anxiety and stress) [1,31], athlete status (e.g., training load, fatigue, and recovery) [31,32,33], and contextual factors (e.g., training or game settings and inciting events) [3,31] interact and influence an athlete’s injury risk. Furthermore, studies suggest that the most prominent injury risk factor is having suffered a previous injury [32,33]. Considering these diverse risk factors, MF might be seen as one of many aspects to take into account when evaluating an athlete’s injury risk. Although the findings from the current preliminary study do not reveal an association, we propose undertaking further research to build on these conclusions.

To explain our findings, it is important to describe the MF phenomenon in the sport context. MF is induced by exposure to demanding cognitive activity [10], which in sports corresponds to the mental demands (i.e., load) imposed by sport-related activities (e.g., training and competition) [34] but also demands derived from being an athlete, such as media and sponsor engagement [16]. To prevent physical and mental fatigue, team sport activities are scheduled by allocating athletes enough time and space to recover physically and mentally [35,36] to guarantee positive training adaptations and optimize readiness and performance [36]. While the detrimental effects of MF on performance have been demonstrated mostly in experimental settings [10], our findings derived from real-world sport practice scenarios, while inducing some elevations in MF, as also previously reported [20,25], revealed that the experienced levels of MF did not relate to subsequent injury risk. Along these lines, MF might affect injury risk more acutely, i.e., in the minutes and hours in which acute MF is present. This is based on findings from experiments in which acute MF was induced with cognitively demanding tests (e.g., the Stroop test) that showed poorer balance, postural control, reaction time and decision-making when acute MF is present [11,22,23]. Differently, in this study, injuries were monitored in the following days and weeks after MF data collection, so the data might not reflect the hypothesized relationships between MF and injury occurrence in acute settings. Future studies should consider monitoring MF more frequently (i.e., daily), and shortly before training sessions and games, in order to better capture potential associations with injuries. Furthermore, in one of the few ecological studies on MF in team sports, Joseph et al. [25] found that higher subjective ratings of MF led to a reduction in running intensity in elite Australian Football. They observed that athletes modified their physical intensity when MF increased as a pacing strategy to prioritize running intensity in decisive competition scenarios. While we did not measure physical intensity in the current study, it is possible that the athletes in this study might have also decreased their physical intensity when mentally fatigued, leading to fewer high-risk scenarios (e.g., collisions, high-intensity jumps and pivoting actions, and coming in proximity to opponents) [2,3], ultimately avoiding the risk of involvement in injuries. Thus, the lack of association between MF and injuries in this study may be explained by the athletes’ self-protective behavior adjustments, as supported by Joseph et al. [25]. Future studies should concurrently collect physical metrics (e.g., external load and intensity) using wearable devices to explore the mediating or moderating effects of MF, exercise intensity, and injury risk.

Another main finding of this study was the limited injury occurrence (7.40 per 1000 h of exposure), with the figure being lower than what was found in previous studies in elite basketball settings (12.6–21.7 injuries per 1000 h) [2,3,6]. As we monitored tier 3 [24] semiprofessional athletes who competed in an official interregional competition organized by the national basketball federation, the training requirements and performance caliber were competitive (Tier 3) but did not match those pertaining to professional athletes. This is an important factor to consider, as injury occurrence in basketball players is typically higher at higher competitive standards [2,3]. In fact, the physical demands of basketball increase in higher-level categories, with greater running, high-intensity, and intermittent demands in professional leagues [4]. These greater physical demands possibly underpin the higher injury risk that is evident at professional levels. In contrast, in the current study, at a medium level of competition, injury risk was considerably lower. Furthermore, another reason for the low injury occurrence found may be the partial monitoring of the season (21 weeks). This low number of injuries might have decreased the statistical power of this study, making it impossible to find small associations that might have cropped up over time. Future studies should monitor MF and injury occurrence throughout full basketball seasons to better explore the phenomenon.

This study has some limitations, namely, the limited sample (a single team) and monitoring window, the dearth of injury events, and the injury risk factors/confounders considered. Future studies should consider monitoring mental load alongside MF and injuries to see if the mental demands have an association with injury risk, as fatigue can be seen as the difference between the imposed load minus the athletes’ capacity to tolerate that load and the recovery practices implemented. Studies should also evaluate the association between MF and injury occurrence among female basketball players as well as at higher competitive levels. Additionally, as MF has been shown to acutely impact athlete’s behaviors, future studies should examine whether MF levels influence injury occurrence over shorter time frames than investigated in this study, e.g., in the following minutes or hours after data collection. In fact, more frequent monitoring of MF (e.g., daily) could improve the robustness of the findings. Finally, it would be beneficial to monitor further variables alongside MF, such as previous injuries, physical qualities, training load, and psychological and contextual factors [1,31,32,33], to gain a more comprehensive understanding.

## 5. Conclusions

Practitioners working with team sport athletes are suggested to address injury occurrence from a multifactorial, non-linear perspective [31]. As our results did not show an association between subjective MF and injury occurrence, monitoring of MF and injuries should be performed including other measures (i.e., objective and performance markers) [37] as well as by monitoring injuries in the minutes and hours immediately following MF evaluation.

In conclusion, this preliminary study on male semiprofessional basketball did not detect a clear association between players’ perceptions of MF and subsequent injury risk in the days leading to MF assessment and up to 4 weeks afterward. While this novel exploration and its findings provide initial insights, the timing of the MF evaluations (only twice per week and on rest days) and the limited sample size limit further generalization of this conclusion. The demonstrated efficacy of an ecologically valid research approach to exploring mental fatigue and injury occurrence provides a foundation for future work.

## Figures and Tables

**Table 1:** Results regarding the effects of mental fatigue on subsequent injury risk.

	Akaike Information Criterion	R^2^ Marginal	Observed Injuries (*n*)	Estimate (95% CI)	OR (95% CI)	z	*p*	Holm-Adjusted Target α Level
Within 1 day	21.380	0.24	2	0.183 (−0.188–0.683)	1.28 (0.82–1.98)	1.11	0.266	0.013
Within 3 days	16.770	0.08	5	0.183 (−0.106–0.472)	1.20 (0.90–1.60)	1.24	0.214	0.008
Within 5 days	18.139	0.02	6	0.174 (−0.003–0.442)	1.19 (0.91–1.56)	1.28	0.202	0.007
Subsequent week	56.200	0.01	8	0.007 (−0.081–0.096)	1.00 (0.89–1.10)	0.16	0.870	0.050
Subsequent 2 weeks	55.000	0.07	6	0.022 (−0.032–0.076)	1.02 (0.97–1.08)	0.80	0.427	0.025
Subsequent 3 weeks	52.610	0.09	6	0.021 (−0.028–0.069)	1.02 (0.97–1.07)	0.83	0.409	0.017
Subsequent 4 weeks	31.200	0.06	3	0.046 (−0.034–0.127)	1.05 (0.97–1.14)	1.14	0.254	0.001

Odds ratio (OR) > 1 indicates that MF increases injury risk; OR < 1 indicates that MF decreases injury risk.

## Data Availability

Data will be made available upon reasonable request.

## Abbreviations

The following abbreviations are used in this manuscript:

MF	Mental fatigue
MD	Match day
